# Cracking the code: unveiling the specific and shared mechanisms behind musculoskeletal interventions

**DOI:** 10.1186/s40945-023-00168-3

**Published:** 2023-07-06

**Authors:** Amy W. McDevitt, Bryan O’Halloran, Chad E. Cook

**Affiliations:** 1 Department of Physical Medicine and Rehabilitation, University of Colorado, Anschutz Medical Campus, CO Aurora, USA; 2 Department of Physical Therapy, School of Health Sciences, Saint Joseph’s University, PA Philadelphia, USA; 3 Department of Orthopaedic Surgery, Duke University, Durham, NC USA; 4 Department of Population Health Sciences, Duke University, Durham, NC USA; 5 Duke Clinical Research Institute, Duke University, Durham, NC USA

**Keywords:** Manual therapy research, Mechanisms of manual therapy, Physiotherapy

## Abstract

**Background:**

Mechanisms reflect the steps or processes through which an intervention unfolds and produces change in a specified outcome variable. Mechanisms are responsible for determining “how treatments work” which has emerged as a critical question for both developing theory and enhancing treatment efficacy. Studies that evaluate “how” treatments work, not just “if” treatments work are of considerable importance.

**Discussion:**

Specific and shared mechanisms research is a promising approach which aims to improve patient outcomes by tailoring treatments to the specific needs of each patient. Mechanisms research is an underexplored area of research requiring a unique research design.

**Conclusion:**

Although mechanisms research is still in its infancy, prioritizing the study of the mechanisms behind manual therapy interventions can provide valuable insight into optimizing patient outcomes.

## Background

The purpose of this viewpoint is to charge researchers with the mission of actively exploring the gap in specific and shared mechanisms research. In February of 2023, Burns and colleagues [1] published a comparative mechanisms study, which evaluated the specific and shared effects of cognitive therapy (CT), mindfulness-based stress reduction (MBSR) and behavior therapy (BT) for patients with chronic pain. This study highlighted the mechanisms underlying these different therapeutic approaches. Despite each approach having theoretically different or specific mechanisms of action, they found that CT, MBSR, and BT produced similar or shared pre- to post-treatment effects on all mechanism variables. Their findings support the likelihood that shared, over specific mechanisms, were responsible for the measurable improvements observed in the study.

Specific treatment mechanisms refer to the unique features of an intervention and are considered the primary reason the intervention is effective. For example, manual therapy techniques exhibit both peripheral and central influences, and reduce muscle spasm, which leads to pain modulation and improved mobility [2]. Appropriate dosage of resistance exercise is associated with increases in muscle fiber size and neural adaptations, which leads to improved strength and endurance [3]. By their nature, specific mechanisms should provide consistent, predictable responses when applied in a therapeutic, efficacious manner.

Shared treatment mechanisms occur when two seemingly different treatments (e.g., manual therapy and resistance exercise) are found to exert their effects on clinical outcomes via similar or common mechanisms (e.g., therapeutic alliance) [4]. Self-efficacy, quality of the therapeutic relationship, and expectancy of treatment benefit were evaluated as potential shared mechanisms [1]. These features were selected because they are not unique to a given treatment but can change with any form of intervention provided. Shared mechanisms may also represent “non-specific” effects, which include all the effects seen in outcomes studies that are not attributed to the specific mechanisms, including placebo, the placebo effect, nocebo, and contextual effects including patient characteristics and expectations, clinician characteristics, the patient-clinician relationship, technique characteristics, and other factors that influence the perceived effectiveness of a given treatment.

To advance our understanding of effective interventions, it is imperative that researchers devote more attention and resources to the study of the specific and shared processes underlying some of our common intervention approaches, including manual therapy. In the following viewpoint we discuss the importance of moving from first order to second order questions to facilitate an improved understanding of the value of medical interventions and their contribution to precision medicine.

### Why is it important to understand mechanisms?

Understanding the mechanisms through which an intervention produces change in outcome variables is critical for advancing precision medicine, which aims to improve patient outcomes by tailoring treatments to the specific needs of each patient. The term “mechanism” reflects the steps or processes through which an intervention (or some independent treatment) unfolds and produces the change in an outcome variable. Mechanisms are responsible for determining “how treatments work”? To move the precision medicine needle, researchers have begun to move past first-order questions, such as “does this treatment work?”, to second order questions such as “how does this treatment work?” Answering if something works (first order), is explored in comparative effectiveness research, and measured via various pre-determined outcomes but limits our knowledge to yes-or-no results without clear pathways for intervention improvement. Asking how something works (second order) has emerged as a more contemporary and critical question for both developing theory and enhancing treatment efficacy [5].

The frequent occurrence of null findings in recent comparative effectiveness research and synthesis-based research may be attributed to our limited understanding of the mechanisms underlying effective interventions. For example, despite numerous studies, there doesn’t seem to be a superior form of exercise for managing chronic low back pain [6]. This is reflected in CLBP guidelines that acknowledge the absence of one particular exercise modality that is superior to others [7]. This finding is not unique to low back pain; several physical therapy treatments (even heavily divergent forms) utilized for painful musculoskeletal conditions end up with comparable patient-reported outcomes such as pain and disability [4]. We need to ask ourselves, why this is the case and why does this occur? Further, would a better understanding of mechanisms foster different outcomes based on clinicians’ choosing an intervention based on its mechanism? It is imperative that we delve deeper into the mechanisms underlying interventions we deem effective, to gain a better understanding of these phenomena.

We propose the following four assertions that affirm the need to move forward in this area of research.

#### Specific and shared mechanisms research will reveal intervention effects more precisely than clinical trials

Within the literature, mechanisms are described by their physiological influences on the body (captured with a blood draw, imaging, or other physiological measure), or by their therapeutic influences (captured with a pen and paper tool-psychological measures or an external device that measures stiffness, range of motion, or movement). Although no single taxonomy for mechanisms exists, reductionist-based mechanistic measures (e.g., changes at the molecular, cellular, or tissue level) have promise in truly defining the physiological influence of a treatment, and are less likely to be influenced by external factors such as context, social, and psychological moderators.

#### Specific and shared mechanisms research is primed to address the physical therapy treatment dosage conundrum

Gaining a better understanding of how treatments work, allows us to refine those treatments and focus more precisely on their active therapeutic components which can significantly improve their efficiency and effectiveness. Specific mechanisms should provide consistent, predictable responses when applied appropriately and in a therapeutic, efficacious manner. Well established treatment approaches such as strengthening exercises require rigorous dosing parameters to increase muscle fiber size and create neural adaptations, which can ultimately lead to improved strength and endurance. To date, there is a dearth of literature demonstrating a relationship between typical forms of clinical treatment approaches, such as exercise, and subsequent morphological changes [8]. Despite thousands of exercise trials, we know very little about the mechanisms of exercise and whether the outcome changes we see are reflective of the specific (morphological or neurophysiological) or shared/non-specific (contextual) effects of exercise.

#### Specific and shared mechanisms research requires a unique research design

A unique study design is necessary to test specific and shared mechanisms involved in an intervention of interest. To facilitate the concurrent evaluation of mechanisms and clinical outcomes, study designs must demonstrate that substantial change in the mechanism is preceded by substantial change in the relevant outcome of interest, while ensuring that the cause precedes the effect. The evaluation of mechanisms has traditionally been confined to preclinical research, lacking comparisons to other interventions, leading to an incomplete understanding of how treatment mechanisms operate in the clinical context. This change in the mechanism must predict the later change in the outcome, further, the change in mechanism is specific to the studied treatment approach. It is crucial to consider that changes in the mechanism have a direct and unique relationship with the outcome, and the timing of measured change aligns with the timing of the application of the technique. Based on this premise, it is essential for study designs to test mechanisms comparing multiple interventions with frequent assessments of response considering both specific and shared mechanisms [9]. Lagged and cross lagged analyses are required to be performed session by session examining both the specific and shared mechanisms. By examining the timing of demonstrated change and ensuring that it is consistent with application of the intervention, we can gain much needed insight into our treatment techniques which ultimately will lead to more effective treatments for our patients [1]^1^.

#### Understanding mechanisms of treatment is an underexplored focus of translational researchers

Approximately 1.71 billion people have musculoskeletal conditions worldwide. The World Health Organization (WHO) states that musculoskeletal disorders are the leading contributor to disability worldwide, with low back pain being the single leading cause of disability in 160 countries. Therefore, elucidating the processes underlying different therapeutic approaches is crucial to advancing our understanding of effective interventions. As a result, the National Institutes of Health (NIH) is explicitly prioritizing opportunities to support research on the mechanisms of action of interventions across multiple professions. The effectiveness, safety and feasibility of physiotherapy interventions have been somewhat elucidated. Once a better understanding of the mechanisms across interventions is reported, the subsequent goal of translational research is to collaborate, fund and design trials that investigate the underlying mechanisms of manual therapy. However, the first step is bench to bedside research to translate knowledge gained from mechanisms studies to clinical interventions. The ultimate objective is to communicate the clinical significance of these findings and establish a connection between research on mechanisms and its application in clinical practice.

### Where do we go from here?

Researchers must study the specific and shared mechanisms behind musculoskeletal interventions. The study of “why” the specific mechanisms of dissimilar interventions lead to similar results is shown to allow researchers to better understand and more closely focus on the common mechanisms that occur concurrently with a treatment’s unique specific effects. Mechanisms research can reduce potential waste in clinical trials, such as studying two treatments with similar mechanisms, only to find similar outcomes. Confirming shared treatment mechanisms across several treatment interventions will improve our options for care, while demystifying why certain treatments are beneficial to certain patient populations. This will allow a greater capacity for targeting interventions toward patient preferences, and perhaps most importantly, answer the conundrum of why seemingly different interventions commonly yield similar outcomes. However, the broader goal of mechanisms research is to guide our focus on implementing purposeful interventions thus contributing towards the advancement of outcomes research and meaningful and lasting patient outcomes.

## Conclusion

Researchers must prioritize mechanisms research to establish verifiable knowns in musculoskeletal care and most effectively contribute towards the advancement of precision medicine and lasting patient outcomes.

## Data Availability

Not available.

## Abbreviations

BT: Behavior therapy
CT: Cognitive therapy
MBSR: Mindfulness-based stress reduction
NIH: National Institutes of Health
WHO: World Health Organization
